# Exploring shared therapeutic targets in diabetic cardiomyopathy and diabetic foot ulcers through bioinformatics analysis

**DOI:** 10.1038/s41598-023-50954-z

**Published:** 2024-01-02

**Authors:** Hanlin Wu, Zheming Yang, Jing Wang, Yuxin Bu, Yani Wang, Kai Xu, Jing Li, Chenghui Yan, Dan Liu, Yaling Han

**Affiliations:** 1 Dalian Medical University, Dalian, 116044 Liaoning Province China; 2 State Key Laboratory of Frigid Zone Cardiovascular Diseases, Department of Cardiology and Cardiovascular Research Institute, General Hospital of Northern Theater Command, Wenhua Road 83, Shenyang, 110016 Liaoning Province China

**Keywords:** Computational biology and bioinformatics, Cardiovascular biology

## Abstract

Advanced diabetic cardiomyopathy (DCM) patients are often accompanied by severe peripheral artery disease. For patients with DCM combined with diabetic foot ulcer (DFU), there are currently no good therapeutic targets and drugs. Here, we investigated the underlying network of molecular actions associated with the occurrence of these two complications. The datasets were downloaded from the Gene Expression Omnibus (GEO) database. We performed enrichment and protein–protein interaction analyses, and screened for hub genes. Construct transcription factors (TFs) and microRNAs regulatory networks for validated hub genes. Finally, drug prediction and molecular docking verification were performed. We identified 299 common differentially expressed genes (DEGs), many of which were involved in inflammation and lipid metabolism. 6 DEGs were identified as hub genes (PPARG, JUN, SLC2A1, CD4, SCARB1 and SERPINE1). These 6 hub genes were associated with inflammation and immune response. We identified 31 common TFs and 2 key miRNAs closely related to hub genes. Interestingly, our study suggested that fenofibrate, a lipid-lowering medication, holds promise as a potential treatment for DCM combined with DFU due to its stable binding to the identified hub genes. Here, we revealed a network involves a common target for DCM and DFU. Understanding these networks and hub genes is pivotal for advancing our comprehension of the multifaceted complications of diabetes and facilitating the development of future therapeutic interventions.

## Introduction

Diabetes mellitus (DM) is a metabolic disease characterized by chronic hyperglycemia^1^. According to the World Health Organization, the number of people with diabetes is expected to reach 693 million by 2045, a > 50% increase from 2017^2^.

Chronic hyperglycemia leads to chronic damage and dysfunction of various tissues, especially heart, brain, kidney, peripheral nerves, eyes and feet^2^. About 4.9 million people worldwide die from diabetes each year, and about 50% of these deaths are due to cardiovascular complications^3^. Diabetic cardiomyopathy (DCM) is one of the most significant cardiovascular complications in patients with diabetes^4,5^. With the rapid increase in the incidence of diabetes, DCM has attracted more and more people's attention. However, unfortunately, since Rubler first proposed DCM in 1972, there has been little progress in the prevention, diagnosis and treatment of DCM in the past 50 years, mainly because the key mechanism of DCM is unclear^6,7^.

Clinically, advanced DCM patients are often accompanied by severe peripheral artery disease, among which diabetic foot ulcer (DFU) is one of the most important extremity peripheral artery diseases^8,9^. Approximately 18.6 million people worldwide are affected by DFU each year^10^. These ulcers precede 80% of lower extremity amputations among people diagnosed with diabetes and are associated with an increased risk of death^10^. Surgical debridement, reducing the pressure of weight bearing on ulcers, treating lower limb ischemia and foot infection are the first-line treatments for DFU^11^. For patients with DCM combined with DFU, there are currently no good therapeutic targets and therapeutic drugs.

The concurrent prevalence of DCM and DFU underscores the interconnectedness of these two complications within the context of diabetes. Accumulating evidence suggests that shared pathological mechanisms, such as chronic inflammation, oxidative stress, and impaired angiogenesis, contribute to the development and progression of both diabetic cardiomyopathy and diabetic foot ulcers^12,13^. Understanding the common molecular pathways and potential therapeutic targets underlying these two conditions is crucial for developing effective interventions that can address multiple diabetes-related complications simultaneously.

In this context, bioinformatics analysis offers a powerful approach to uncovering potential therapeutic targets that may be shared between diabetic cardiomyopathy and diabetic foot ulcers^14^. By integrating and analyzing large-scale biological datasets, bioinformatics methodologies allow for the identification of key molecular players and pathways implicated in the pathogenesis of these complications^15^. The elucidation of common therapeutic targets holds significant promise for advancing the development of novel treatment strategies that can mitigate the burden of diabetic cardiomyopathy and diabetic foot ulcers, ultimately improving the clinical outcomes and quality of life for individuals with diabetes.

**Figure 1:**
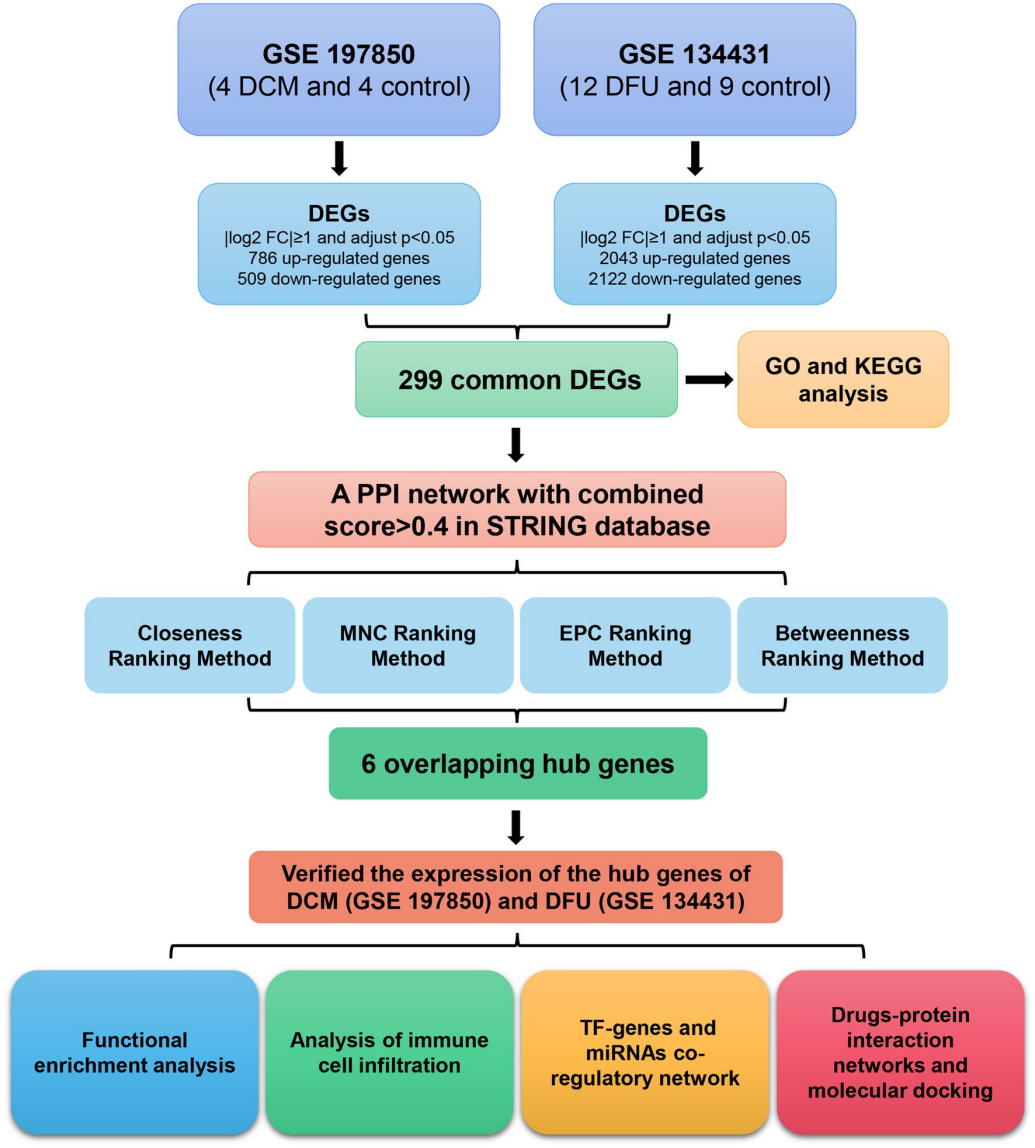
Research design flow chart.

Given the substantial impact of DCM and DFU on public health, as well as the growing prevalence of diabetes globally, investigating the shared therapeutic targets in these two conditions is of utmost importance. This research aims to contribute to a deeper understanding of the molecular underpinnings of diabetic complications and to provide valuable insights for the development of targeted therapies that can address the interconnected nature of DCM and DFU. By shedding light on the commonalities in the pathological processes underlying these conditions, this study seeks to pave the way for innovative and comprehensive approaches to managing diabetes-related complications. The study design was illustrated in a flow diagram (Fig. 1).

## Results

### Identification of DEGs in DCM and DFU

**Figure 2:**
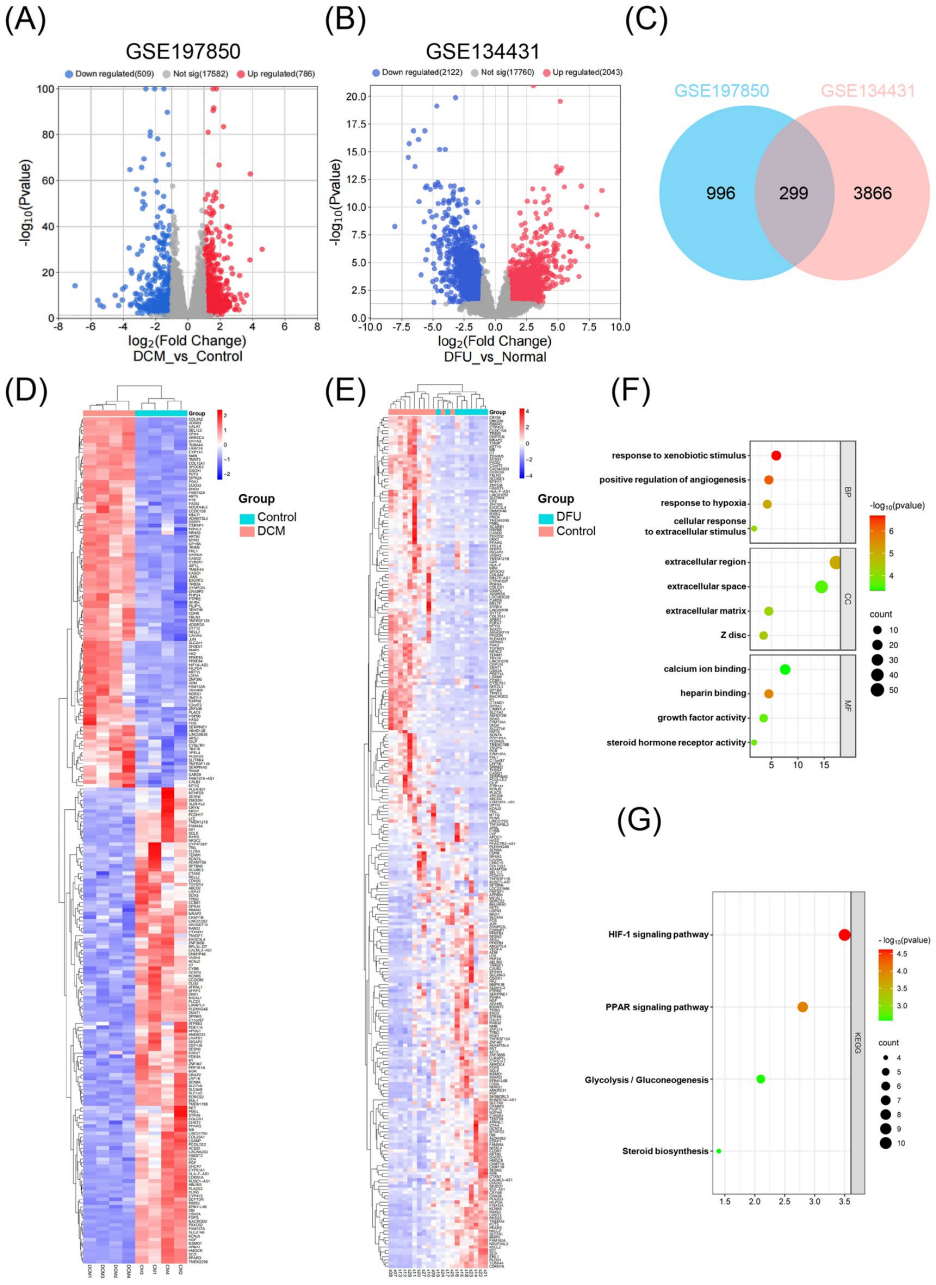
Differentially expressed genes (DEGs) and functional enrichment analysis in DCM and DFU datasets. (**A,B**) Volcano plot of DEGs in GSE197850 and GSE134431 datasets. Red color indicated up-regulated genes and blue color indicated downregulated genes. (**C**) Venn diagram of overlapping DEGs among two GEO datasets. (**D,E**) Heat maps of overlapping DEGs in GSE197850 and GSE134431 datasets. (**F**) Gene ontology (GO) enrichment analysis of 299 overlapping DEGs. (**G**) Kyoto encyclopedia of genes and genomes (KEGG) pathway analysis of 299 overlapping DEGs.

We downloaded the Series GSE 197,850 dataset about DCM and the series GSE 134,431 dataset about DFU from the NCBI GEO database. After screening with the threshold of an adjusted p value < 0.05 and |log_2_FC|> 1.0, there were 1295 DEGs in the GSE 197,850 dataset, among which 786 genes were up-regulated and 509 genes were down-regulated in the DCM samples (Fig. 2A). There were 4165 DEGs in GSE 134,431 dataset, of which 2043 genes were up-regulated and 2122 genes were down-regulated in DFU samples (Fig. 2B). In addition, a Venn diagram analysis was performed to evaluate the common DEGs between GSE 197,850 (DCM) and GSE 134,431 (DFU). As shown in Fig. 2C, 299 overlapping DEGs were identified. The heatmap analyses were used to visualize the overlapping DEGs of the two datasets (GSE 197,850 and GSE 134,431) (Fig. 2D,E). The basic information of common DEGs has been summarized in Supplementary Table S1.

### Enrichment analysis of overlapping DEGs

To analyze the biological functions and pathways involved in 299 common DEGs, GO and KEGG pathway enrichment analyses were performed. After screening with the threshold of an adjusted p value < 0.05, we select the top four significantly enriched GO terms and the top four KEGG terms. In terms of biological processes, DEGs were principally associated with response to xenobiotic stimulus, positive regulation of angiogenesis, response to hypoxia and cellular response to extracellular stimulus. The analysis of cell component indicated that DEGs significantly enriched in extracellular region, extracellular space, extracellular matrix and Z disc. The results of molecular function showed that DEGs were mainly enriched in calcium ion binding, heparin binding, growth factor activity and steroid hormone receptor activity (Fig. 2F, Supplementary Table S2). KEGG pathway analysis of DEGs showed that the top four significant pathways were enriched in HIF-1 signaling pathway, PPAR signaling pathway, glycolysis/gluconeogenesis and steroid biosynthesis (Fig. 2G, Supplementary Table S3).

### PPI network construction and identification of hub genes

**Figure 3:**
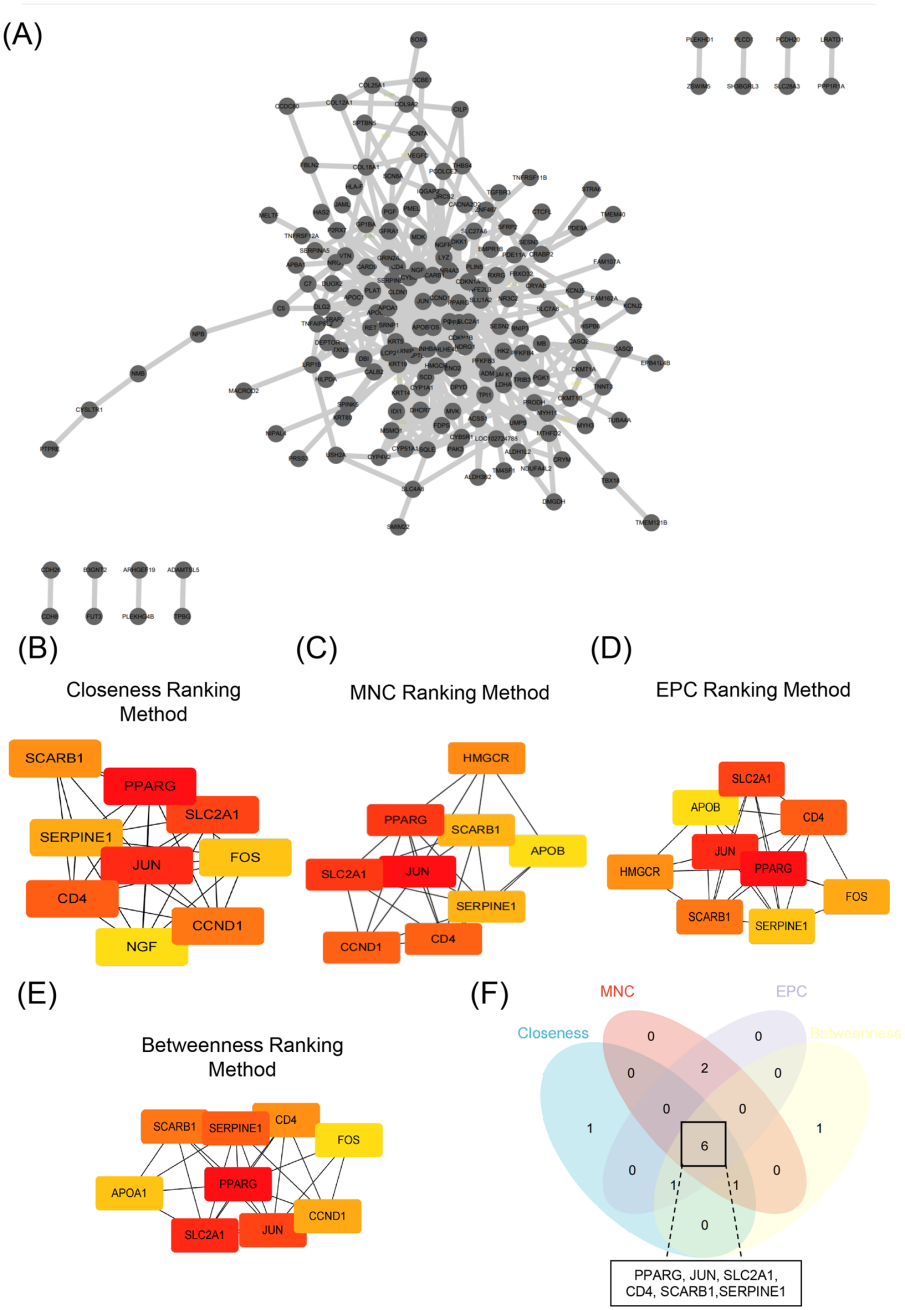
Identification of Hub genes in overlapping DEGs among two GEO datasets. (**A**) Protein–protein interaction of the overlapping DEGs. (**B–E**) The closeness ranking method, the maximal neighborhood component (MNC) ranking method, the edge percolated component (EPC) ranking method, and the betweenness ranking method for hub genes identification. (**F**) Venn diagram for identifying hub genes among different ranking methods.

The PPI network was constructed based on a STRING database to determine the interactions between overlapping DEGs. The obtained results are then imported into Cytoscape software for visual analysis (Fig. 3A). PPI network was analyzed by the closeness ranking method. As shown in Fig. 3B, the top 9 genes were identified as potential hub genes: Activator protein 1 (JUN), Peroxisome proliferator activated receptor gamma (PPARG), Solute carrier family 2 member 1 (SLC2A1), CD4 molecule (CD4), Cyclin D1 (CCND1), Scavenger receptor class B member 1 (SCARB1), Serpin family E member 1 (SERPINE1), Protein C-Fos (FOS), Nerve growth factor (NGF). Based on MNC ranking method, the top 9 genes were identified as potential hub genes: JUN, PPARG, SLC2A1, CD4, CCND1, SCARB1, SERPINE1, 3-hydroxy-3-methylglutaryl-CoA reductase (HMGCR), Apolipoprotein B (APOB) (Fig. 3C). Based on EPC ranking method, the top 9 genes were identified as potential hub genes: JUN, PPARG, SLC2A1, CD4, SCARB1, SERPINE1, HMGCR, FOS, APOB (Fig. 3D). Based on Betweenness ranking method, the top 9 genes were identified as potential hub genes: JUN, PPARG, SLC2A1, CD4, SCARB1, SERPINE1, CCND1, FOS, Apolipoprotein A1 (APOA1) (Fig. 3E). As shown in Fig. 3F, 6 overlapping hub genes were identified: PPARG, JUN, SLC2A1, CD4, SCARB1, SERPINE1.

### The expression of hub genes

**Figure 4:**
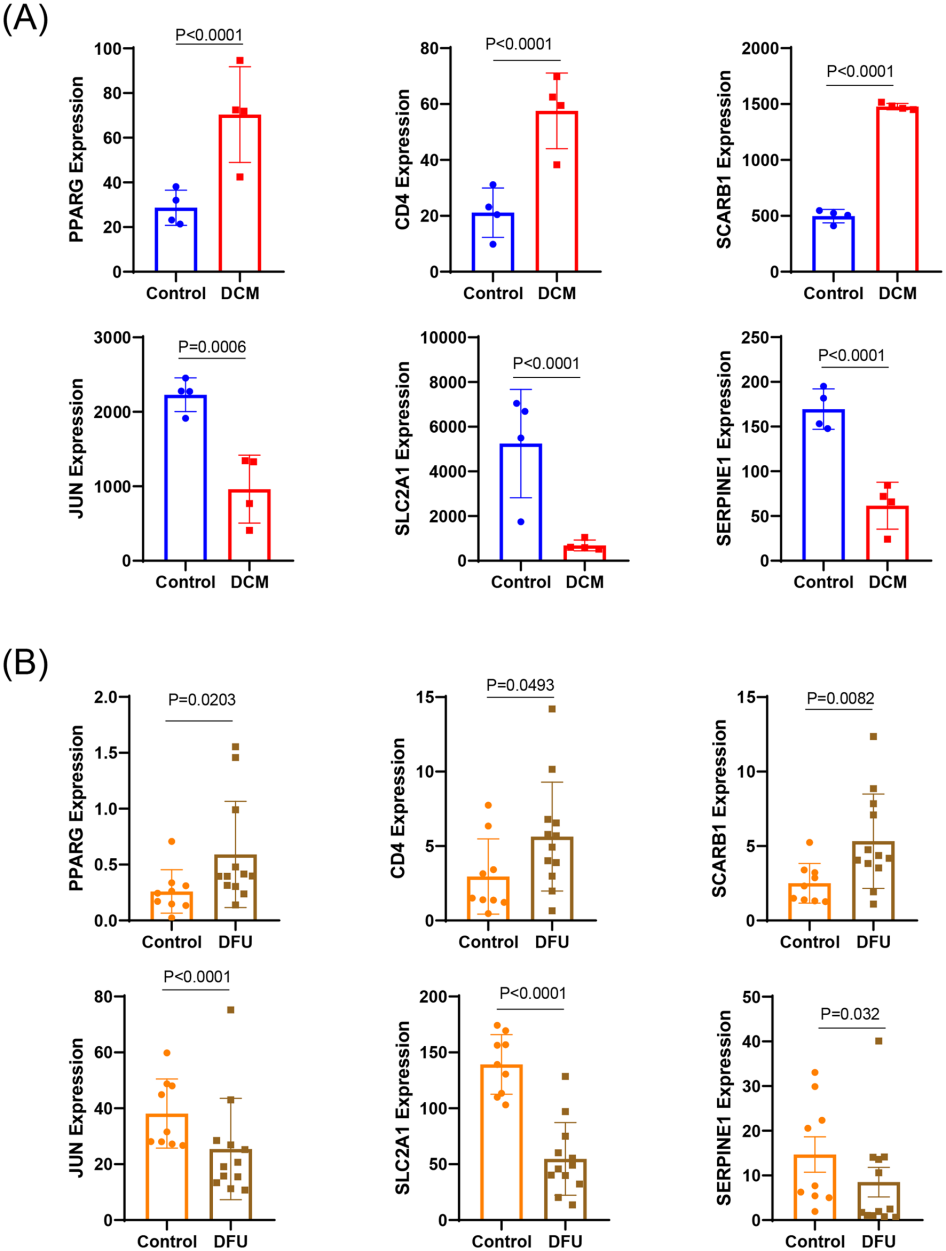
The expression of hub genes in two GEO datasets. (**A**) The mRNA expression of hub genes in CSE197850 datasets. (**B**) The mRNA expression of hub genes in CSE134431 datasets. Data are presented as mean ± SEM.

In order to identify the genes necessary for the co-occurrence of DCM and DFU, we verified the expression of the hub genes of DCM (GSE 197,850, Fig. 4A) and DFU (GSE 134,431, Fig. 4B) from GEO database. According to our results, compared to the control group, three hub genes were significantly enhanced in the DCM and DFU samples: PPARG, CD4 and SCARB1. At the same time, three hub genes were significantly weakened: JUN, SLC2A1 and SERPINE1.

### Functional enrichment analysis of proteins interacting with hub genes

**Figure 5:**
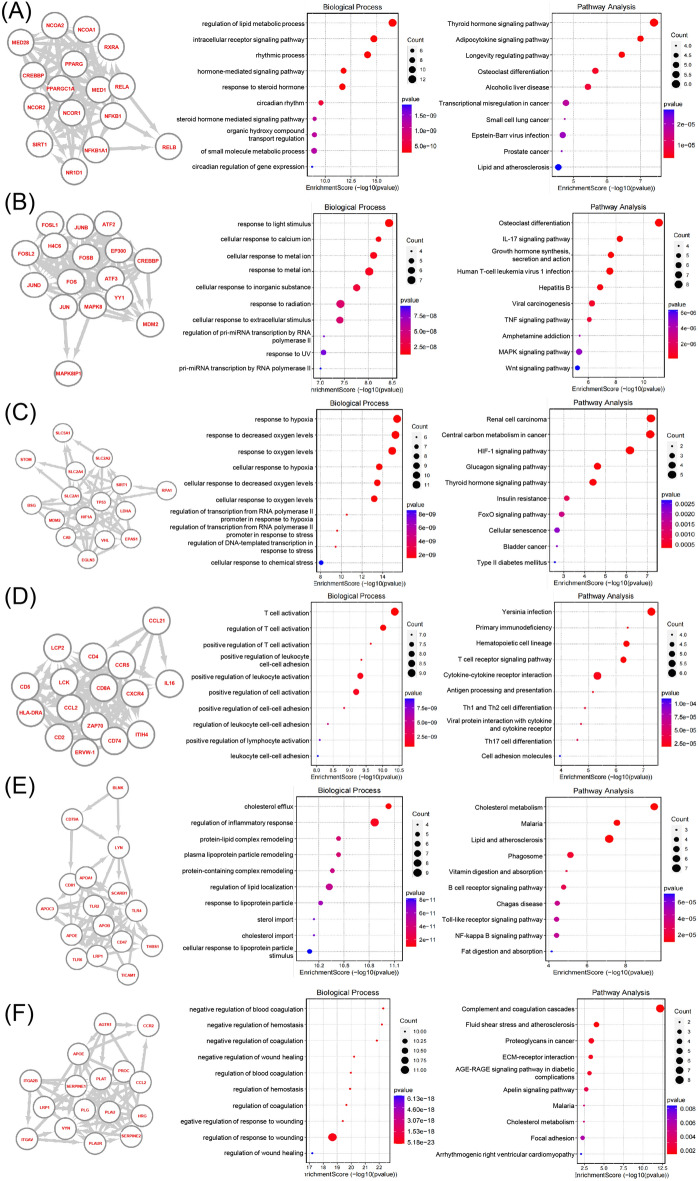
Functional enrichment analysis of proteins interacting with six genes. (**A**) Protein interaction network of PPARG. BP and KEGG enrichment analysis of protein interaction network of PPARG. (**B**) Protein interaction network of JUN. BP and KEGG enrichment analysis of protein interaction network of JUN. (**C**) Protein interaction network of SLC2A1. BP and KEGG enrichment analysis of protein interaction network of SLC2A1. (**D**) Protein interaction network of CD4. BP and KEGG enrichment analysis of protein interaction network of CD4. (**E**) Protein interaction network of SCARB1. BP and KEGG enrichment analysis of protein interaction network of SCARB1. (**F**) Protein interaction network of SERPINE1. BP and KEGG enrichment analysis of protein interaction network of SERPINE1.

In order to clarify the biological functions of each hub gene, functional enrichment analysis of proteins interacting with 6 hub genes was performed. PPARG and its interacting proteins were significantly enriched in the regulation of lipid metabolic biological process (Fig. 5A). JUN-interacting proteins were significantly enriched in cellular response to metal ion biological process and inflammatory response signaling pathway such as IL-17 and human T-cell leukemia pathways (Fig. 5B). SLC2A1-interacting proteins were significantly enriched in response to hypoxia biological process and HIF-1 signaling pathway (Fig. 5C). CD4 and its interacting proteins were widely involved in various T cell functions to influence immunity (Fig. 5D). SCARB1-interacting proteins were significantly enriched in lipid metabolism biological process and inflammatory response signaling pathway (Fig. 5E). SERPINE1-interacting proteins were significantly enriched in negative regulation of blood coagulation and wound healing biological process (Fig. 5F). Therefore, these hub genes were widely involved in lipid metabolic, response to metal ion, inflammatory response, hypoxia, blood coagulation and wound healing biological process and related signaling pathways.

### Differential expression analysis of immune cell infiltration

**Figure 6:**
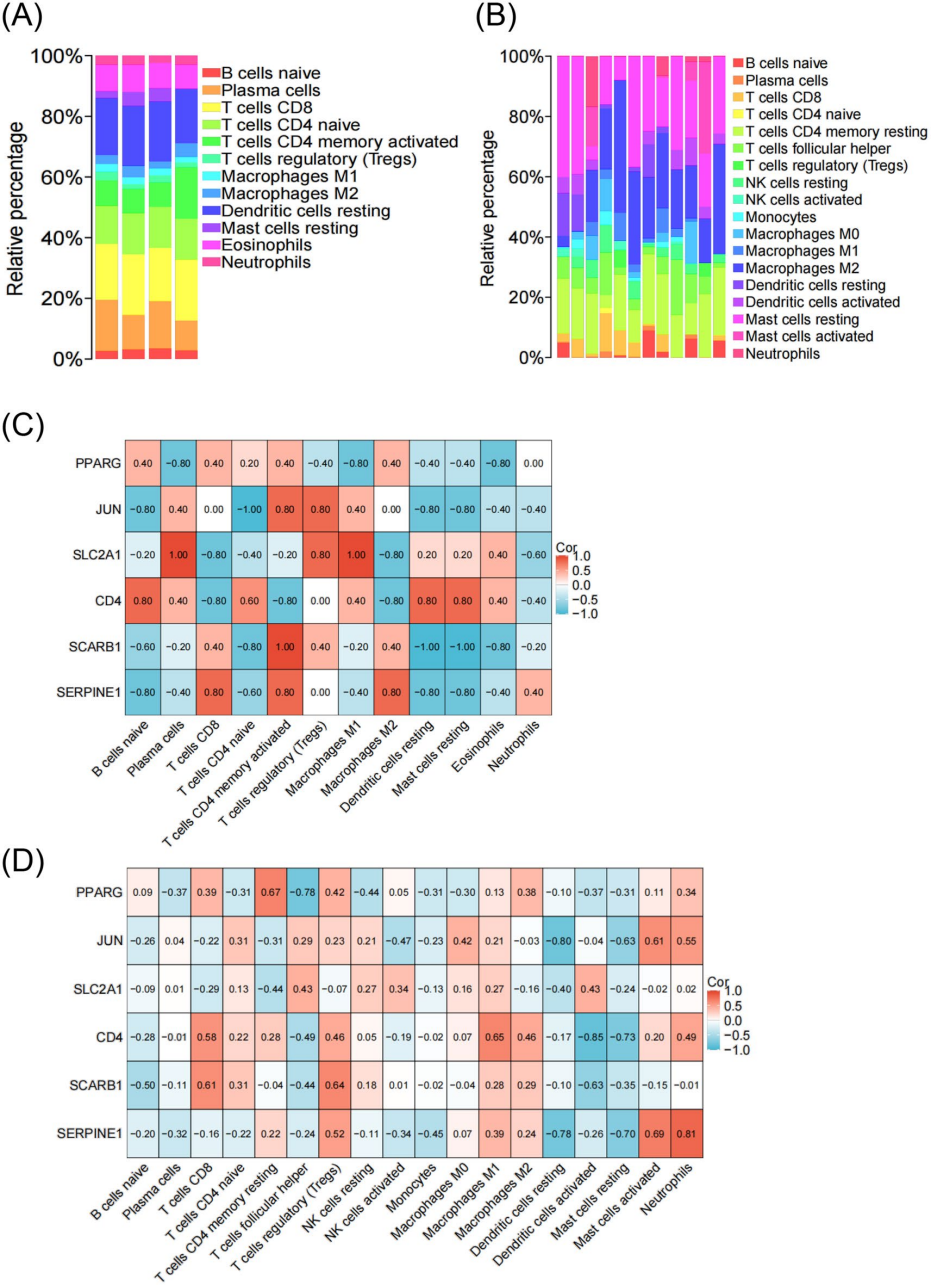
DCM and DFU are associated with immune infiltration. (**A**) The relative abundance of distinct immune cells subsets in DCM. (**B**) The relative abundance of distinct immune cells subsets in DFU. (**C**) The correlation between immune cell infiltration and the expression of PPARG, JUN, SLC2A1, CD4, SCARB1 and SERPINE1 in DCM cohort. (**D**) The correlation between immune cell infiltration and the expression of PPARG, JUN, SLC2A1, CD4, SCARB1 and SERPINE1 in DFU cohort.

Inflammation plays an important role in the occurrence and progression of DCM and DFU^16,17^. To assess the extent of inflammation, we employed the CIBERSORT algorithm to estimate the degree of immune cell infiltration in patients diagnosed with DCM and DFU. The relative abundance analysis of different immune cell subsets in DCM showed that T cells were the main subsets of infiltrating immune cells (Fig. 6A). In DFU, T cell subsets and macrophage subsets are the main subsets of infiltrating immune cells (Fig. 6B). In the DCM T cells cohort, SLC2A1 and CD4 were significantly negatively correlated with CD8 T cells, while SERPINE1 was significantly positively correlated. There was a significant negative correlation between JUN and SCARB1 in CD4 naive T cells. In CD4 memory activated T cells, JUN, SCARB1 and SERPINE1 were significantly positively correlated, while CD4 was significantly negatively correlated. JUN and SCARB1 were significantly positively correlated with T cells regulatory (Tregs, Fig. 6C). In the DFU T cells cohort, CD4 and SCARB1 were positively correlated with CD8 T cells. PPARG was positively correlated with CD4 memory resting T cells, but negatively correlated with T cells follicular helper. SCARB1 and SERPINE1 were positively correlated with Tregs. In the DFU macrophage cohort, CD4 was positively correlated with M1-type macrophages (Fig. 6D).

In DCM and DFU, correlation coefficient analysis revealed a significant correlation between the hub genes and the level of immune cell infiltration (Fig. 6C,D). This showed that DCM and DFU were associated with immune infiltration.

### Construct transcription factor (TF)-gene and miRNA regulatory network

**Figure 7:**
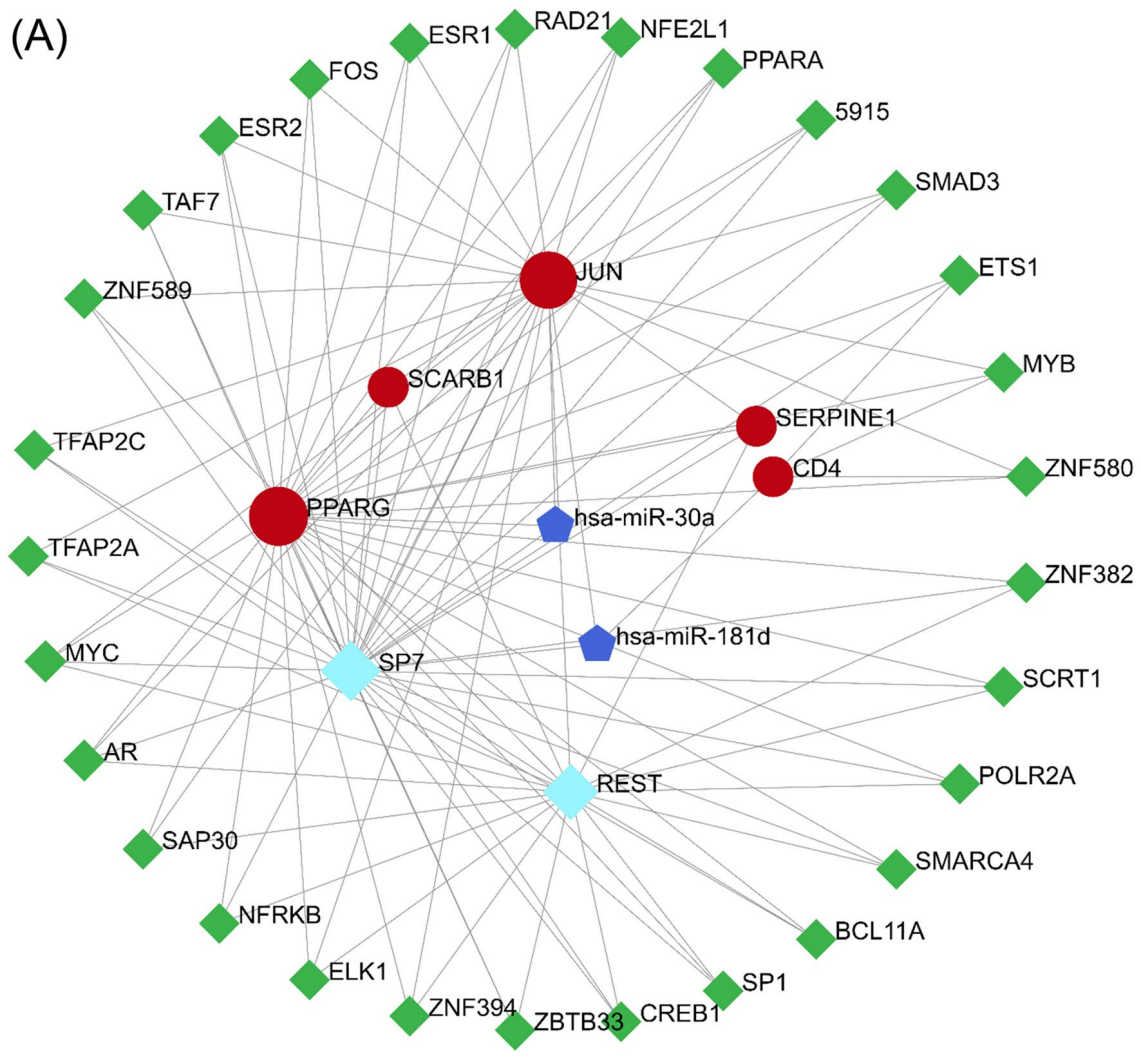
Construct transcription factor (TF)-gene and miRNA regulatory network. (**A**) TFs and microRNAs regulatory networks for validated hub genes. Red: hub genes, green: TF genes, wathet blue: key TF genes, blue: miRNAs.

Next, we used Networkanalyst to predict the association between TF-genes, miRNAs and hub genes. We combined the TF-Knock v2.0 database with the miRTarBase screening subnetwork to validate our predicted TF-genes + miRNAs network. We screened the potential transcription factors of hub genes "PPARG", "JUN", "SLC2A1", "CD4", "SCARB1" and "SERPINE1" from the TF-Knock database. In the miRTarBase database, we screened potential miRNAs that were experimentally confirmed by western blot, clip-seq, micro-array, etc. Our network reveals 38 nodes, 106 edges, and 5 seed genes (Fig. 7A). Transcription factors SP7, REST, miR-30a and miR-181d may play key regulatory roles in the regulation of hub genes.

### Identification of candidate drugs and targeted chemical interactions in DCM and DFU

**Figure 8:**
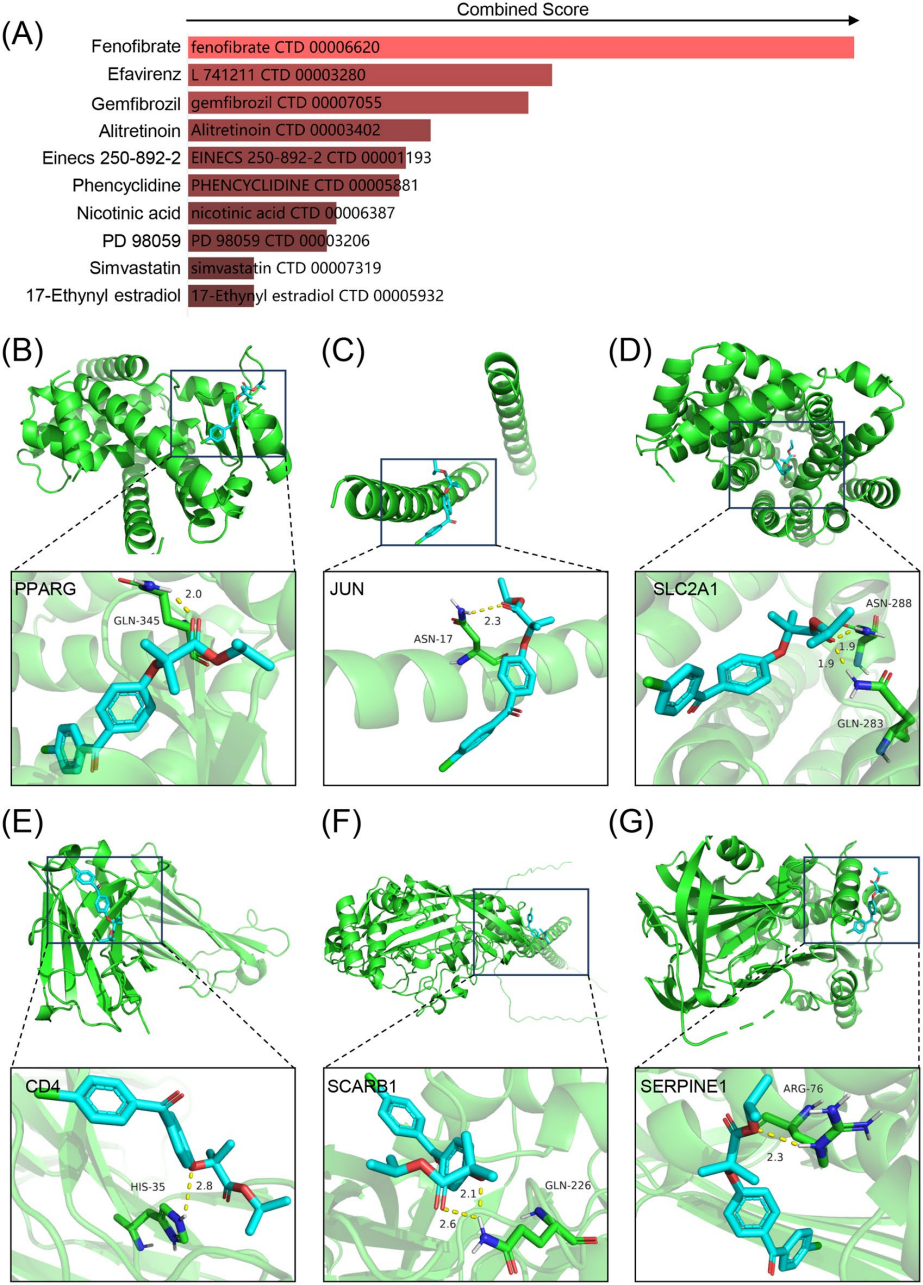
Identification of candidate drugs and molecular docking patterns. (**A**) The combined score of interactions between known molecules and hub genes in the DSigDB database. (**B-G**) Molecular docking mode of fenofibrate and PPARG (**B**), JUN (**C**), SLC2A1 (**D**), CD4 (**E**), SCARB1 (**F**) and SERPINE1 (**G**).

Chemical-protein interaction networks are important research tools for understanding protein function and advancing drug discovery. The hub genes of DCM and DFU were used to identify drug candidates based on DSigDB database enrichment. The top 10 drug molecules were selected based on their P-values as potential drug candidates. The 10 drug candidates included Fenofibrate, Efavirenz, Gemfibrozil, Alitretinoin, Einecs 250-892-2, Phencyclidine, Nicotinic acid, PD 98,059, Simvastatin and 17-Ethynyl estradiol (Fig. 8A). We summarized the drug candidates (top 10) adjusted P-value by gene-drug interaction enrichment analysis in Supplementary Table S4. These drug candidates reacted with the hub gene, indicating that they might be suitable for treating both diseases.

Of all the drug candidates, fenofibrate had the highest combined score (Fig. 8A). In addition, molecular docking predicted the binding of fenofibrate to hub genes: PPARG, JUN, SLC2A1, CD4, SCARB1 and SERPINE1. Molecular docking results are shown in Fig. 8B–G. Interestingly, fenofibrate had low stabilization energy at its binding sites with six target proteins and formed stable structures. The binding energy, number and location of hydrogen bond formation of fenofibrate and 6 hub genes were evaluated by AutoDock calculation, as shown in Supplementary Table S5. Therefore, fenofibrate might have the potential to treat DCM combined with DFU.

## Discussion

In our study, through the screening of DEGs and the construction of PPI network, we found 6 hub genes that may have significant differences in the pathogenesis and development of DCM and DFU: PPARG, JUN, SLC2A1, CD4, SCARB1 and SERPINE1.

PPARG encodes PPAR-γ, a member of the nuclear receptor peroxisome proliferator activating receptor (PPAR) subfamily, which is a regulator of adipocyte differentiation. PPARG directs cardiac energy metabolism in cardiomyocytes, thereby affecting pathological heart failure and diabetic cardiomyopathy^18^. PPARG activation effectively improved the foot withdrawal threshold, protected the sciatic nerve myelin structure, and improved the intraepidermal nerve fiber density of foot skin^19^. These studies show that PPARG has a regulatory role in both DCM and DFU, which is consistent with the results predicted by our biological analysis.

JUN is a protein that is highly similar to a viral protein and interacts directly with specific target DNA sequences to regulate gene expression. Although rarely reported in DCM and DFU. However, our analysis results showed that JUN was significantly correlated with DCM and DFU. This may be related to JUN's regulation of inflammation and lipid metabolism^20,21^.

SLC2A1 promotes glucose transporters, which are responsible for constitutive or basal glucose uptake^22,23^. It has a very broad substrate specificity and can transport a variety of aldoses, including pentose and hexose^24^. Our results showed that SCL2A1 was significantly down-regulated in both DCM and DFU patients. It may be a key therapeutic target for DCM combined with DFU.

CD4 encodes the CD4 membrane glycoprotein of T lymphocytes, which plays a crucial role in the immune response^25^. Previous studies have shown that T lymphocyte depletion improves cardiac fibrosis in streptozotocin induced diabetic cardiomyopathy^26^. CD4^+^ T cells affect DFU healing by regulating FOXP3, providing a new direction for future treatment of DFU^27^. We found that CD4 was significantly elevated in DCM and DFU, and CD4 was found to promote T cell activation through functional enrichment. The regulation of T cell activation by CD4 may have important significance for the treatment of DCM combined with DFU.

The protein encoded by SCARB1 is a plasma membrane receptor for high-density lipoprotein cholesterol (HDL)^28^. SCARB1 mediates the transfer between cholesterol and high-density lipoprotein plays an important role in lipid homeostasis in type 2 diabetes^28^. And genetic variants of SCARB1 can lead to severe early-onset coronary artery disease^29^. In our study, SCARB1, as a hub gene, was significantly increased in both DCM and DFU. This suggests that SCARB1 may be involved in diabetes-related complications by regulating lipid homeostasis.

SERPINE1 encodes plasminogen activator inhibitor-1 (PAI-1), a downstream target of heart-transforming growth factor β, which is associated with cardiac fibrosis^30^. Studies showed that a hyperosmotic, autosomal recessive cardiac fibrosis phenotype was found in young adults with homozygous migration variants of SERPINE1, suggesting that cardiac homeostasis requires an optimal range of levels of PAI-1^30^. PAI-1 is elevated in obese people with type 2 diabetes and may contribute to increased cardiovascular disease risk independently of traditional factors^31^. We screened SERPINE1 as a key target gene that was significantly elevated in both DCM and DFU. These results suggest that SERPINE1 may be involved in the fibrotic process that leads to multiple complications of diabetes.

We used GSEA to reveal 6 identified central genes widely associated with adaptive immune responses. Patients with DCM and DFU may benefit from anti-inflammatory therapy in the microenvironment^32,33^. However, these genes were not work together on the same immune cell type. Therefore, interventions targeting single-cell immune responses may not be effective against DCM combined with DFU. We also used Networkanalyst to predict the association between TF-genes, miRNAs and hub genes. Transcription factors SP7, REST, miR-30a and miR-181d may be the key regulatory targets of hub gene, providing a new perspective for the treatment of DCM patients with DFU.

In recent years, the combined analysis of metabolomics, lipidomics and proteomics has been widely used in the analysis of potential drug targets^34–36^. Sodium-glucose cotransporter 2 (SGLT 2) inhibitors are the gold standard in the treatment of type 2 diabetes. Among them, empagliflozin (EMPA) has been shown to have a beneficial effect on heart failure^34^. Multi-omics analysis showed that EMPA could regulate and partially restore the levels of multiple metabolites related to stress in cardiomyocytes under high glucose environment, alleviating lipid toxicity^34^. Enhanced diabetic cardiac fatty acid (FA) metabolism and perturbations in the biosynthesis of unsaturated fatty acid and arachidonic acid metabolism are potential drivers of the cardiovascular benefits of EMPA^35^. Animal experiments have also demonstrated that fatty acid oxidation is one of the targets of EMPA therapy in db/db mouse hearts^36^. These results suggest that improving lipid metabolism may be an effective treatment strategy to alleviate multiple complications of diabetes.

Fenofibrate is primarily used as a lipid-lowering drug to reduce cholesterol levels in patients at risk of cardiovascular disease^37^. Fenofibrate reduces low density lipoprotein (LDL), very low-density lipoprotein (VLDL) and triglyceride (TG) levels, and increases high density lipoprotein (HDL) levels^38^. In people with high triglyceride levels, fenofibrate plus statin treatment was associated with lower all-cause death and cardiovascular disease^37^. In a multi-country randomized controlled trial involving 9795 people aged 50–75 years with type 2 diabetes, fenofibrate reduced total cardiovascular events^39^. Fenofibrate rescues diabetes-associated ischemia-mediated angiogenesis impairment by mercaptopurine independent regulation of thioredoxin interactions^40^. Our study shows that fenofibrate reacts with hub genes. Molecular docking also showed that fenofibrate and 6 hub genes could form hydrogen bonds to form stable structures. This suggests that fenofibrate may be a potential therapeutic agent for advanced DCM combined with DFU. Recent experimental studies have demonstrated that fenofibrate, as a peroxisome proliferator-activated receptor (PPAR) agonist, protects corneal nerves against diabetic eye disease (DR)^41^. Fenofibrate up-regulated Nrf2 inhibits diabetes-related ferroptosis and delays the progression of diabetic nephropathy (DN)^42^. These results suggest that fenofibrate may have a broad therapeutic effect in patients with multiple complications of diabetes.

Our review of the published literature showed that there was limited research on common therapeutic targets and drugs between DCM and DFU, especially bioinformatic analyses. Here, we screened overlapping DEGs, hub genes, and TFs of DCM and DFU to elucidate the common targets of DCM and DFU. However, our study has some limitations. First, our work needs to be further verified by experiments in vivo and in vitro. Secondly, the function of hub genes needs further experimental exploration, which is the focus of our upcoming research.

In summary, our study offers compelling evidence suggesting that specific hub genes may mediate the common pathogenesis of DCM and DFU. The identification of six hub genes—PPARG, JUN, SLC2A1, CD4, SCARB1, and SERPINE1—highlights their potential involvement in the pathophysiology of these conditions. Furthermore, our findings indicate that fenofibrate, known for its lipid-lowering effects, may hold therapeutic promise for patients with DCM and concurrent DFU. Our findings provided a possible direction to explore common therapeutic targets and drugs for DCM and DFU. Despite the need for further validation and exploration, these findings offer valuable insights that could potentially pave the way for the development of novel treatment strategies targeting both conditions simultaneously.

## Materials and methods

### Dataset download

Use the search term "diabetic cardiomyopathy" and "diabetic foot ulcer", we screened the whole Gene Expression Omnibus (GEO) database (http://www.ncbi.nlm.nih.gov/geo/)^43^. The following data sets were incorporated into the analysis: First, the archival information should include case and control groups. Second, the genome was sequenced. Third, these datasets must provide raw data that can be further analyzed. Based on the GPL11154 Illumina HiSeq 2000 (Homo sapiens) platform and the GPL18573 Illumina NextSeq 500 (Homo sapiens) platform collected two gene datasets (GSE 197,850, GSE 134,431) for DCM, DFU and control group from the GEO database. The GSE 197,850 dataset included gene expression profiles from 4 DCM samples and 4 normal controls. The GSE 134,431 dataset included 12 DFU patients and 9 controls.

### Screening of differentially expressed genes (DEGs)

Using GEO 2R online analytical tools (https://www.ncbi.nlm.nih.gov/geo/geo2r/) extraction and analysis of DEGs respectively, the tool is GEO database contained in the web application based on R language. The p values were corrected by Benjamini and Hochberg method to reduce the false positive rate. The threshold of DEGs screening was |log_2_ FC|≥ 1 and adjust p < 0.05. Use the R packages "complexheatmap" and "ggplot 2" to visualize the DEG obtained from the two datasets to generate heat maps and volcano maps, respectively. In addition, the overlapping DEGs between DCM and DFU were delineated using the Venn diagrams with the Venn online platform (http://bioinformatics.psb.ugent.be/webtools/Venn/). These overlapping DEGs are used for subsequent analysis.

### GO and KEGG analyses of DEGs

The above overlapping DEGs were analyzed for Gene ontology (GO) functional enrichment, consisting of biological processes (BP), cell components (CC), and molecular functions (MF), as well as Kyoto encyclopedia of genes and genomes (KEGG) signaling pathway enrichment using the R package "clusterProfiler"^44^. Significance was considered when the adjusted p < 0.05^45–47^.

### Protein–protein interaction establishment and identification of hub genes

To further explore the interactions between the common genes obtained above, a PPI network was constructed using the search tool for interacting genes (STRING) (http://string-db.org/)^48^. A minimum interaction score of above 0.4 was considered to be significant. The PPI network was then visualized using Cytoscape software. Then, we use the Cytoscape plugin Minimal Common Oncology Data Elements (MCODE, http://apps.cytoscape.org/ apps/mcode) to select key protein expression. Then, four algorithms were applied to screen the hub genes with high connectivity in PPI network.

### Verification of hub gene expression

We confirmed the transcriptional levels of the above hub genes in GSE197850 (DCM) and GSE134431 (DFU). We used t-test for inter-group assessment P < 0.05.

### Immune infiltration analysis

CIBERSORT algorithm was used to analyze the gene expression matrix to estimate the infiltrating immune cell subpopulation in the sample. The samples were filtered with an adjusted P-value < 0.05, and the outputs of the MOABS algorithm and the immune cell infiltration matrix were calculated. Use R package "GSVA" for single sample GSEA analysis (ssGSEA) and use R package "pheatmap" (https://CRAN.R-project.org/package=pheatmap) to visualize correlations between samples.

### Transcription factor (TF)-genes and miRNAs regulatory network

Through the Networkanalyst platform (https://www.networkanalyst.ca/) to generate the TF-genes and miRNAs regulation network. TF-genes through the TF-Knock2.0 database (https://bio.liclab.net/KnockTFv2/search.php) for screening and validation. MiRNAs were filtered and verified by miRTarBase (https://mirtarbase.cuhk.edu.cn/~miRTarBase/), which binding has been experimentally confirmed by western blot, clip-seq, micro-array, and other methods.

### Potential drug evaluation and molecular docking

Analysis was performed using the Drug Characteristics Database (DSigDB)^49^. The crystal structures of these key proteins are available from the Protein Database (https://www.rcsb.org/). All docking experiments were performed using the Autodock tool (version 1.5.4). The result was expressed by binding energy. The final image was presented by Pymol Molecular Visualization System 2020.

### Supplementary Information

Supplementary Information.

## Data Availability

All data generated or analysed during this study are included in this published article.

## Abbreviations

DCM: Diabetic cardiomyopathy
DFU: Diabetic foot ulcer
GEO: Gene expression omnibus
TF: Transcription factor
DEGs: Differentially expressed genes
DM: Diabetes mellitus
KEGG: Kyoto encyclopedia of genes and genomes
GO: Gene ontology
BP: Biological processes
CC: Cell components
MF: Molecular functions
