# Supplementary material for: Exploring shared therapeutic targets in diabetic cardiomyopathy and diabetic foot ulcers through bioinformatics analysis
Source: Sci Rep. 2024 Jan 2;14:230. doi: 10.1038/s41598-023-50954-z (PMC10761883; doi:10.1038/s41598-023-50954-z)
Supplement: Supplementary file 1 — Supplementary Information. [file 41598_2023_50954_MOESM1_ESM.docx]

**Exploring shared therapeutic targets in diabetic cardiomyopathy and diabetic foot ulcers through bioinformatics analysis**

Hanlin Wu^a,b#^, Dan Liu^b#^, Zheming Yang^b^, Jing Wang^b^, Yuxin Bu^b^, Yani Wang^b^, Kai Xu^b^, Chenghui Yan^b^, Yaling Han^b*^

^a^ Dalian Medical University, Dalian, Liaoning Province, 116044, China

^b^ State Key Laboratory of Frigid Zone Cardiovascular Diseases, Department of Cardiology and Cardiovascular Research Institute, General Hospital of Northern Theater Command, Shenyang, Liaoning Province, 110016, China

^#^These authors contributed equally to this work.

*Corresponding author:

Yaling Han, State Key Laboratory of Frigid Zone Cardiovascular Diseases, Cardiovascular Research Institute and Department of Cardiology, General Hospital of Northern Theater Command, Wenhua Road 83, 110016 Shenyang, China. E-mail: hanyaling@163.net

**Supplementary Tables**

**Supplementary Table S1.** Basic information for common DEGs

| **Gene symbol** | **log2FC (GSE197850)** | **log2FC (GSE134431)** |
| --- | --- | --- |
| ABCD2 | 1.1881388 | 2.5611556 |
| ABHD12B | -1.6093453 | 1.8653901 |
| ABLIM3 | 1.5765512 | -2.2436731 |
| ACSS1 | 1.8949647 | 1.2688422 |
| ADAM9 | -1.1810883 | -1.0089642 |
| ADAMTS6 | 1.3570527 | -1.2009086 |
| ADAMTSL5 | -1.1133558 | -2.0816226 |
| ADGRG5 | -2.6796461 | 1.5702612 |
| ADM | -1.2546147 | -3.1554949 |
| AIF1L | -1.0859886 | -1.5081898 |
| ALDH1L2 | 1.394757 | -1.5435563 |
| ALDH3B2 | -1.2191605 | -1.2987974 |
| ANGPTL4 | -1.4269058 | -1.619815 |
| ANKRD31 | 1.3539551 | -3.0573433 |
| APBA1 | 1.1986582 | 1.1146216 |
| APOC1 | 1.3631494 | 2.7475884 |
| ARHGEF19 | 1.2525416 | 1.2164178 |
| ARRDC4 | -1.5473903 | -1.5109086 |
| ARSJ | -1.2144739 | -1.9877326 |
| ATP8B3 | 1.0811981 | -1.3370341 |
| ATRNL1 | 1.1690556 | -3.2891912 |
| B3GNT2 | -1.1503461 | -1.6137373 |
| BHLHE40 | -1.3019161 | -1.3425319 |
| BMPR1B | 1.1558689 | -1.0224676 |
| BNIP3 | -1.4748701 | -3.2997432 |
| C11orf87 | 4.5853125 | 2.3636677 |
| C2orf72 | -1.7537966 | 2.1542067 |
| C7 | 1.7273622 | 4.5513377 |
| CACNA2D2 | 1.1171227 | 1.4133024 |
| CALB2 | -1.8504595 | -4.5214364 |
| CALML3-AS1 | 1.9414533 | -2.6650774 |
| CARD9 | -1.0621085 | 1.5470917 |
| CASQ1 | -1.2264595 | 1.2711301 |
| CASQ2 | -1.8687329 | 2.7950177 |
| CAVIN4 | -1.0075698 | -2.0522627 |
| CCBE1 | 1.9078584 | 1.9866369 |
| CCDC158 | -1.9606299 | 2.622313 |
| CCDC80 | 1.2289098 | 1.8602091 |
| CCN3 | 1.0839022 | 2.340286 |
| CD4 | 1.3615196 | 1.1692791 |
| CDH26 | 1.7112141 | -3.1668139 |
| CDH8 | -1.3705378 | -1.5127642 |
| CDKN1A | 1.1059099 | -2.8048881 |
| CEP126 | 1.0962109 | 1.34565 |
| CHST2 | 1.2439211 | -2.8229143 |
| CILP | -1.2436508 | 5.0246997 |
| CKMT1A | 1.1634025 | -1.1919214 |
| CKMT1B | 1.2739791 | -1.1080847 |
| CLDN1 | 1.2116319 | -1.4264502 |
| COL12A1 | -2.7285178 | -1.6913019 |
| COL25A1 | 1.8661809 | 3.6305641 |
| COL9A2 | -2.8579832 | 2.2274887 |
| COLCA1 | 2.3768885 | 1.7429246 |
| CPA4 | -3.6344035 | -1.5260761 |
| CPZ | 1.4979147 | 1.6161198 |
| CRABP2 | -1.0552324 | -1.629824 |
| CRYAB | -1.1418791 | -2.2571926 |
| CRYM | 1.243534 | 1.8307202 |
| CSRNP1 | -1.073335 | -1.7020878 |
| CTXN1 | 1.0748071 | -2.0450987 |
| CTXND1 | 1.4937839 | 2.9716218 |
| CYB5R1 | -1.0053144 | -1.3135225 |
| CYBB | 1.0633612 | 2.0837047 |
| CYP1A1 | -1.0760749 | 3.4313352 |
| CYP4F35P | 1.9412471 | 2.5245082 |
| CYP4V2 | 1.23967 | 1.5691546 |
| CYP51A1 | 1.2600478 | -1.2492637 |
| CYSLTR1 | -1.396452 | 2.073698 |
| DBI | 1.236881 | -1.4438629 |
| DEPTOR | 1.841928 | 1.5325401 |
| DHCR7 | 1.1274383 | -1.2495695 |
| DIPK2A | -1.0370771 | -1.2459569 |
| DKK1 | 1.5669956 | 3.7501119 |
| DLG2 | 1.1482096 | 2.6358395 |
| DMGDH | 1.7635879 | 2.0733016 |
| DNM1P46 | 1.4482658 | 1.9183086 |
| DPYD | 1.1571583 | 1.082109 |
| DUOX2 | -2.3139008 | -1.3963051 |
| EML1 | 1.0342929 | -2.8212244 |
| ENO2 | -2.7129616 | -1.3699338 |
| EPB41L4B | 1.215101 | -1.5024212 |
| ESRP1 | -1.3497784 | -1.2872465 |
| EXOC3L4 | 1.1312813 | 3.0091006 |
| FAM107A | 2.4645501 | 1.5788226 |
| FAM153A | -2.3842812 | 4.8612109 |
| FAM162A | -1.2356058 | -2.7636748 |
| FAM167A-AS1 | -1.501761 | 2.1241628 |
| FAM43A | 1.110126 | -1.4442106 |
| FAM88E | 1.1095631 | 1.7880307 |
| FAM88F | 1.857397 | 2.9477883 |
| FAM89A | 1.0180978 | -2.2097571 |
| FBLN2 | -2.1019781 | 1.4237198 |
| FBXO32 | 1.082362 | 1.5449184 |
| FDPS | 1.5620233 | -1.2578554 |
| FHL1 | -1.948406 | 1.3997821 |
| FILIP1L | -1.4779525 | -1.910574 |
| FOS | -1.0157 | -1.4858217 |
| FUT3 | -2.0455162 | -2.5514858 |
| GALK1 | -1.7100555 | -1.4109456 |
| GCNT4 | 1.588841 | -1.4847473 |
| GFRA1 | 1.4033572 | 5.2681374 |
| GP1BA | -1.0697137 | 1.4181551 |
| GRAP2 | 2.5696724 | 1.2234754 |
| GRIN2A | -1.4410432 | 2.2543937 |
| H19 | -1.5361946 | -3.2911695 |
| HAS2 | -1.2523102 | 1.5714772 |
| HIF1A-AS3 | -2.8247497 | -2.1014127 |
| HILPDA | -1.5039911 | -1.8980768 |
| HILPDA-AS1 | -1.130288 | -1.2881386 |
| HK2 | -2.275916 | -3.8242066 |
| HLA-F | 1.4519583 | 1.2751151 |
| HLA-F-AS1 | 2.3358743 | 1.5480286 |
| HMGCR | 1.5024842 | -1.7896286 |
| HOTS | -1.4471289 | -3.3680522 |
| HS6ST3 | 1.5529858 | 3.5830284 |
| HSPB6 | -1.9781362 | 2.31881 |
| IDI1 | 1.247891 | -2.6514363 |
| IDI2-AS1 | 1.1651573 | -2.4961715 |
| INHBA | -1.1398235 | -1.4763842 |
| IQGAP2 | 2.3958788 | 1.7355504 |
| JAML | -1.3101026 | 1.6905616 |
| JUN | -1.207311 | -1.5669783 |
| KCNJ2 | 2.3999983 | 1.1539751 |
| KCNJ5 | 1.2470573 | 1.3938202 |
| KCNK6 | 1.1849689 | -1.5364149 |
| KRT19 | -1.2917164 | -1.5820534 |
| KRT5 | -5.4331376 | -1.1229326 |
| KRT80 | -3.5325046 | -2.1590876 |
| KY | 1.5818575 | 3.9048969 |
| L3MBTL4 | 1.2055661 | 2.072354 |
| LDHA | -2.5209618 | -1.4145053 |
| LINC00638 | -1.3884232 | 1.5412655 |
| LINC01252 | 1.4797449 | 1.3617012 |
| LINC01279 | 1.1546069 | 2.2045963 |
| LINC01750 | 1.3204996 | 1.3002753 |
| LOC100996720 | 1.0029633 | 1.5327698 |
| LOC101928274 | 1.2069361 | 1.4932281 |
| LOC101928383 | -1.1771511 | 2.1714395 |
| LOC101930496 | -2.92485 | -2.4003284 |
| LOC102723458 | 1.2148463 | 1.4827882 |
| LOC102724682 | 1.0086742 | -2.3824236 |
| LOC102724788 | 1.2804625 | 1.4732482 |
| LOC102724852 | -1.5204941 | -3.2923649 |
| LOC105370114 | -1.042622 | 2.537818 |
| LOC105371030 | 1.279146 | 2.1570134 |
| LOC105371480 | 1.8708235 | -1.4457113 |
| LOC105371486 | 1.1150486 | 1.6776348 |
| LOC105374085 | 1.2350014 | 1.3226852 |
| LOC105374985 | 1.2704972 | -1.6852375 |
| LOC105375721 | 1.3638257 | 1.942726 |
| LOC105378675 | 1.2240938 | -3.151195 |
| LOC105378936 | 1.9220296 | 1.8189197 |
| LOC105379003 | 1.2216058 | -1.7457163 |
| LOC107985303 | -1.1748776 | -1.92401 |
| LOC107985656 | 1.0955378 | 1.2971434 |
| LOC107986289 | 1.5338334 | 2.1221955 |
| LOC112267876 | -1.5316232 | -3.5976485 |
| LOC112268022 | 1.2550801 | 2.0530229 |
| LOC112268474 | -1.0046974 | 1.4081544 |
| LOC221946 | -2.0212251 | -2.1657477 |
| LOC283028 | 1.617758 | 1.5232609 |
| LRATD1 | 2.4065048 | -1.297183 |
| LRP1B | 1.4172442 | 2.5011223 |
| LRRC15 | -2.8328612 | -1.2366528 |
| LSAMP | 1.6026778 | 1.0888135 |
| LURAP1L | 1.6253355 | -1.0346585 |
| LYZ | 1.8365868 | 1.2527937 |
| MACROD2 | 1.7443671 | 2.2653141 |
| MB | 1.1822004 | 2.283138 |
| MDK | 1.1185544 | 1.7036922 |
| MELTF | -2.0393762 | 2.5402242 |
| MELTF-AS1 | -1.207651 | 1.7712045 |
| MICAL1 | 1.0371777 | -1.0424539 |
| MIR675 | -1.4729992 | -3.0546895 |
| MRAP2 | 1.6881925 | 1.2757799 |
| MSMO1 | 1.5368052 | -1.8413127 |
| MT1G | -2.3762302 | 2.7875067 |
| MTHFD2 | 1.3117954 | -1.1110304 |
| MYH11 | -1.2604752 | 1.6557818 |
| MYH3 | -1.9603817 | 1.1232226 |
| NDRG1 | -2.0845037 | -2.0002346 |
| NDUFA4L2 | -3.1559962 | -2.1735259 |
| NELL2 | -1.060461 | -2.0650835 |
| NFE2L3 | -1.0131817 | 1.5075096 |
| NGF | 3.3055816 | -1.8771964 |
| NIPAL4 | -1.1397349 | -2.1103805 |
| NMB | -2.6008747 | -1.1658173 |
| NPB | 1.5957461 | -2.9281493 |
| NR3C2 | 1.1999322 | 2.5175506 |
| NR4A3 | -1.2432277 | -1.9092057 |
| NRG1 | 1.92903 | -1.4738713 |
| NXPH4 | -1.092894 | -1.5682906 |
| P2RX7 | 1.428413 | 1.0060852 |
| PADI2 | -1.227882 | 2.5298028 |
| PAK3 | 1.7574088 | 2.5935782 |
| PCDH17 | 1.462245 | -1.6872165 |
| PCDH20 | -1.7968992 | 4.7083842 |
| PCOLCE2 | 1.111723 | 2.6171281 |
| PDE11A | 1.2190126 | 2.7314043 |
| PDE9A | 1.0678677 | 1.3067674 |
| PFKFB3 | -2.9579724 | -1.9931745 |
| PFKFB4 | -2.6751558 | -2.2711118 |
| PGF | 1.6014304 | -2.4247772 |
| PGK1 | -1.1758436 | -1.1097887 |
| PGR | 1.322875 | 1.7131121 |
| PHACTR2-AS1 | 1.4671661 | -1.2636198 |
| PHF24 | -1.1875694 | -1.0536426 |
| PLA2G3 | 2.4080414 | -3.0518165 |
| PLAC9 | -1.5679528 | 1.69938 |
| PLCD1 | 1.5270725 | -2.265784 |
| PLEKHD1 | 1.3932283 | 1.4726499 |
| PLEKHG4B | 1.5219489 | -1.5820555 |
| PLIN5 | 1.3078858 | 3.0111574 |
| PMEL | 1.4455164 | 3.2438729 |
| PMFBP1 | 1.1634271 | -1.3517709 |
| PNCK | -1.0223425 | 3.3188712 |
| PPARD | 1.2217592 | -2.0292134 |
| PPARG | 1.0097276 | 1.319114 |
| PPP1R1A | 1.0336772 | 3.1278565 |
| PRODH | 1.2094518 | 1.2122395 |
| PRSS3 | -1.0058396 | -2.5253323 |
| PTPRE | -1.2942229 | -1.4110401 |
| QSOX1 | -1.121916 | -3.1392741 |
| RAB32 | 1.0583538 | -1.7725816 |
| RBM43 | 1.0476406 | 1.2816599 |
| RELL2 | 1.0300684 | -2.3081101 |
| RET | 1.3863478 | -2.6225559 |
| RIMS3 | 1.266705 | -2.5773405 |
| RPL34-DT | 1.6370776 | 3.1715418 |
| RUNDC3A-AS1 | -1.2198973 | -1.2380607 |
| RUSC1-AS1 | 1.5954021 | -1.1616582 |
| RXRG | 1.7721988 | 3.0886112 |
| SAXO1 | 1.9380615 | 1.0692333 |
| SCARB1 | -1.1778202 | 1.0856754 |
| SCD | 1.2159792 | -3.5853049 |
| SCN7A | 1.930915 | 6.0413786 |
| SCN8A | 1.109736 | -1.6699805 |
| SCUBE3 | 1.076963 | 1.3010274 |
| SEL1L3 | -1.3599717 | -1.0951346 |
| SERPINA5 | -1.3919803 | 2.8664914 |
| SERPINE1 | -1.4727283 | -1.9286914 |
| SESN2 | 1.9117805 | -2.2496488 |
| SESN3 | 1.5103437 | -1.1933841 |
| SFRP2 | 1.211792 | 1.4611044 |
| SGMS2 | -1.0054605 | -1.268952 |
| SH3BGRL3 | -1.1042615 | -1.9105688 |
| SH3D21 | -1.3531958 | -2.0964417 |
| SLC1A2 | 1.0447147 | 2.4313225 |
| SLC27A6 | 1.3432506 | 3.6597756 |
| SLC28A3 | 1.0085297 | -2.2584605 |
| SLC2A1 | -2.9127627 | -1.8892638 |
| SLC4A8 | 1.4485462 | -1.4653289 |
| SLC7A8 | 1.2621777 | -1.1045915 |
| SLITRK4 | -1.4139517 | 2.3014614 |
| SMIM22 | -1.0188298 | 1.6776994 |
| SORCS2 | 1.1424388 | -1.6476504 |
| SOX5 | 1.1623017 | 2.7097632 |
| SPINK5 | 1.0911572 | -2.999367 |
| SPOCK2 | -1.7217233 | 1.0409292 |
| SPTBN5 | 1.1652328 | -1.4145204 |
| SQLE | 1.289839 | -1.2585196 |
| STRA6 | 1.0984774 | -1.9959449 |
| SYNE4 | 1.0847025 | 1.3374625 |
| SYNPO2L | -1.0860188 | -3.5991363 |
| SYT12 | -1.9864278 | 1.6401621 |
| TBX18 | -1.1158843 | 1.413711 |
| TENM1 | 1.9582658 | 3.211914 |
| TENT5B | -1.4537458 | -1.8445113 |
| TGFBR3 | 1.5818749 | 1.6122356 |
| THBS4 | -1.0176334 | 2.1253683 |
| TM4SF1 | 1.165868 | -2.7571232 |
| TMEM121B | 1.243608 | 2.2311804 |
| TMEM178B | 1.623095 | 2.6143393 |
| TMEM229B | 2.4521985 | 1.675044 |
| TMEM40 | -1.1802703 | -2.3888918 |
| TNFAIP8L2 | 1.0853265 | 1.1216487 |
| TNFRSF11B | -1.1401784 | -2.2101664 |
| TNFRSF12A | -2.1580496 | -2.3844518 |
| TNNT3 | -6.9772938 | 1.739186 |
| TPBG | 1.3411664 | -1.3160905 |
| TRIB3 | 2.0990164 | -1.0950807 |
| TRIL | 2.4816889 | 3.0156522 |
| TRIM9 | -1.8750406 | 1.3700346 |
| TUBA4A | -1.7355689 | -2.3958145 |
| TXNIP | -1.2237346 | 2.3620386 |
| USH2A | 1.923241 | 1.7630681 |
| USP43 | 2.3916684 | -1.759557 |
| VASH2 | 1.735125 | 1.2318411 |
| VEGFA | -1.0706288 | -1.8044233 |
| VEGFD | 1.0494265 | 3.5685971 |
| YPEL4 | -1.2228999 | 1.093542 |
| ZMAT1 | 1.0258304 | 1.9090017 |
| ZNF208 | 1.4245611 | 2.0645994 |
| ZNF215 | -1.5469276 | -1.5223538 |
| ZNF385B | 1.1409031 | -2.8474913 |
| ZNF395 | -1.1536252 | 1.0460129 |
| ZNF467 | 1.4535293 | -1.2796523 |
| ZNF536 | -1.15304 | 3.4940404 |
| ZSWIM5 | -1.6790182 | 1.3275672 |

**Supplementary Table S2.** GO enrichment analysis of common DEGs

| **Category** | **Term** | **Count** | **Adjusted P-value** |
| --- | --- | --- | --- |
| GOTERM_BP_DIRECT | response to xenobiotic stimulus | 17 | 2.10E-07 |
| GOTERM_BP_DIRECT | positive regulation of angiogenesis | 13 | 6.80E-07 |
| GOTERM_BP_DIRECT | response to hypoxia | 12 | 8.70E-06 |
| GOTERM_BP_DIRECT | cellular response to extracellular stimulus | 5 | 6.40E-05 |
| GOTERM_CC_DIRECT | extracellular region | 50 | 9.50E-06 |
| GOTERM_CC_DIRECT | Z disc | 10 | 3.40E-05 |
| GOTERM_CC_DIRECT | extracellular matrix | 13 | 6.10E-05 |
| GOTERM_CC_DIRECT | extracellular space | 42 | 2.70E-04 |
| GOTERM_MF_DIRECT | heparin binding | 13 | 2.40E-06 |
| GOTERM_MF_DIRECT | steroid hormone receptor activity | 5 | 1.70E-04 |
| GOTERM_MF_DIRECT | growth factor activity | 10 | 2.30E-04 |
| GOTERM_MF_DIRECT | calcium ion binding | 22 | 4.60E-04 |

**Supplementary Table S3.** KEGG enrichment analysis of common DEGs

| **Category** | **Term** | **Count** | **Adjusted P-value** |
| --- | --- | --- | --- |
| KEGG_PATHWAY | HIF-1 signaling pathway | 10 | 2.40E-05 |
| KEGG_PATHWAY | PPAR signaling pathway | 8 | 9.20E-05 |
| KEGG_PATHWAY | Glycolysis / Gluconeogenesis | 6 | 2.60E-03 |
| KEGG_PATHWAY | Steroid biosynthesis | 4 | 2.70E-03 |

**Supplementary Table S4.** Candidate drugs (top 10) identified from gene–drug interaction enrichment analysis

| **Term** | **Adjusted P-value** |
| --- | --- |
| Fenofibrate | 6.93E-06 |
| Rac Efavirenz | 3.30E-05 |
| Gemfibrozil | 3.30E-05 |
| Alitretinoin | 4.73E-05 |
| Einecs 250-892-2 | 4.73E-05 |
| Phencyclidine | 4.73E-05 |
| Nicotinic acid | 6.42E-05 |
| PD 98059 | 6.42E-05 |
| Simvastatin | 8.99E-05 |
| 17-Ethynyl estradiol | 8.99E-05 |

**Supplementary Table S5.** The binding energies for key drug targets were evaluated through AutoDock calculations

| **Drug targets** | **Binding energy** | **Hydrogen bond** | **Binding site** |
| --- | --- | --- | --- |
| PPARG_Fenofibrate | -7.4 | 1 | Gln-345 |
| JUN_Fenofibrate | -5.4 | 1 | Asn-17 |
| SLC2A1_Fenofibrate | -8.7 | 2 | Gln-283, Asn-288 |
| CD4_Fenofibrate | -6.6 | 1 | His-35 |
| SCARB1_Fenofibrate | -6.9 | 2 | Gln-226 |
| SERPINE1_Fenofibrate | -7.2 | 1 | Arg-76 |
